# The inclusive fitness controversy: finding a way forward

**DOI:** 10.1098/rsos.170335

**Published:** 2017-07-19

**Authors:** Jonathan Birch

**Affiliations:** Department of Philosophy, Logic and Scientific Method, London School of Economics and Political Science, Houghton Street, London, WC2A 2AE, UK

**Keywords:** social evolution, inclusive fitness, Hamilton's rule, kin selection, natural selection, adaptation

## Abstract

This paper attempts to reconcile critics and defenders of inclusive fitness by constructing a synthesis that does justice to the insights of both. I argue that criticisms of the regression-based version of Hamilton's rule, although they undermine its use for predictive purposes, do not undermine its use as an organizing framework for social evolution research. I argue that the assumptions underlying the concept of inclusive fitness, conceived as a causal property of an individual organism, are unlikely to be exactly true in real populations, but they are approximately true given a specific type of weak selection that Hamilton took, on independent grounds, to be responsible for the cumulative assembly of complex adaptation. Finally, I reflect on the uses and limitations of ‘design thinking’ in social evolution research.

## 1. Introduction

The debate about the foundations of inclusive fitness theory that has followed in the wake of Nowak, Tarnita & Wilson's [1] critique has been remarkably polarizing. After several rounds of rebuttals and replies, there is still little evidence of any serious reconciliation between the theory's critics [2–10] and its defenders [11–24]. It doesn't have to be this way. I believe that, on the main points of disagreement, it is possible to find a way forward that does justice to the insights of both camps. My aim in this paper is to find that way forward.

## 2. Kin selection, Hamilton's rule and inclusive fitness

The concepts of kin selection, Hamilton's rule and inclusive fitness are often run together. They are, to be sure, closely related, but they should be distinguished [21,25]. Here is how I will use these concepts in this paper.

### 2.1. Kin selection

*Kin selection* is a process that occurs in nature: a variety of natural selection in which the direction of evolutionary change is affected by correlated interaction between genetic relatives. It is something that happens out there in the world, independently of the methods social evolution theorists may invent to analyse it, and independently of the controversies theorists may have about these methods [17,23,26].

I will not discuss the empirical evidence for kin selection in this paper [12,13,20]. Ultimately, I do not think the current controversies surrounding inclusive fitness and related ideas are primarily empirical debates about the existence, or otherwise, of kin selection. Both sides accept that kin selection occurs [27,28]. There are empirical disagreements about the importance of kin selection in explaining particular biological phenomena, such as the evolution of eusociality [8,14,22], but I do not see these debates about particular biological phenomena as the core of the controversy. At its core, this is a controversy not about the existence of kin selection, but about the explanatory value of the conceptual and theoretical framework Hamilton [29–32] constructed to make sense of it. This framework has two key ingredients: Hamilton's rule and inclusive fitness.

### 2.2. Hamilton's rule

*Hamilton's rule* is a mathematical condition for positive change in the frequency of a trait in a population undergoing natural selection, expressed in terms of three population statistics defined with reference to that trait: relatedness (*r*), benefit (*b*) and cost (*c*). The rule states that a trait will undergo positive change due to natural selection if and only if *rb* − *c* > 0 [29].

In its most general, regression-based form [17,33], the rule is a highly abstract result that applies not only to cases of kin selection in the above sense but to all cases of natural selection, including those cases in which *r* = 0 and those cases in which *r* > 0 due to causes, such as ‘greenbeard’ mechanisms [34,35], that do not rely on correlated interaction between biological kin in the ordinary sense of the word. Because of its highly abstract nature, there is room for legitimate debate as to what, if anything, Hamilton's rule explains about social evolution, and I will consider this question in §3.

### 2.3. Inclusive fitness

*Inclusive fitness*, as Hamilton [30] conceived it, is a property of an individual organism, defined as a weighted sum of the effects on reproductive success it causes by means of its behaviour. The weights are coefficients of relatedness. The term has sometimes taken on other meanings, and other authors prefer to use the term ‘inclusive fitness’ to refer to a property of a trait, strategy or lineage [36–38]. But for Hamilton, it was a property of an individual organism (see §4).

It is, at first sight, a strange quantity (see §4). Yet, Hamilton regarded it as the best way of thinking about the fitness of an organism in the context of social evolution, and his successors in what we might call the Hamiltonian tradition in social evolution theory, such as Grafen, Gardner and West, tend to agree [39–41]. They claim inclusive fitness is uniquely able to capture the *design objective* of social adaptation—the goal towards which all social adaptation is directed. I consider this claim in §§4 and 5.

## 3. The status of Hamilton's rule

### 3.1. What the critics get right

There are various formulations of Hamilton's rule, and the different versions attach different meanings to the cost, benefit and relatedness coefficients [23,42,43]. The current controversy has predominantly centred on the generalized, regression-based version first formulated by Queller [33], in which relatedness is a regression coefficient capturing the statistical association between the genotypes of social partners, and cost and benefit are partial regression coefficients in a regression model of reproductive success. I have elsewhere called this version HRG (G for both *general* and *genic*) [42].

Critics of HRG point out, correctly, that the rule in this generalized, regression-based form is general because it is tautology-like [2,7]. The rule says, roughly speaking, that the evolutionary change under one, coarse-grained description is positive if and only if the evolutionary change under another, finer-grained description is also positive. The coarse-grained description is simply the overall change in the frequency of a gene, or in the mean value of a polygenic character. The finer-grained description uses a regression model to partition the overall change into an *rb* component that captures indirect fitness effects and a−*c* component that captures direct fitness effects. The rule simply notes an equivalence between two different ways of describing change, without making any detailed assumptions about the causes of change.

By way of analogy, it is akin to saying that a candidate wins a US presidential election if and only if that candidate wins more than 269 votes in the electoral college: the result, described in general terms, is equivalent to the result described at a finer grain of analysis [4]. This is why (although a few assumptions are required) Hamilton's rule can be said to be ‘as general as the genetical theory of natural selection itself’ [11].

As a consequence, HRG is not much use for prediction, as Allen *et al.* [7] correctly note. If one has the information necessary to calculate the relatedness, cost and benefit coefficients in HRG exactly (i.e. complete information about the genotypic values, fitness values and social interactions in the target population), then one also has the information necessary to calculate the exact response to selection directly. Prediction does not come into it. The change under one, finer-grained description cannot be said to predict the change under another, coarser-grained description, any more than a state-by-state breakdown of an election result can be said to predict the overall result.

Alternative versions of Hamilton's rule invoke stronger assumptions, and these versions, not HRG, should be used for predictive purposes. In particular, an approximate, ‘marginal’ version that replaces the partial regression coefficients with partial derivatives of a fitness function can be used to derive predictions about evolutionary stable strategies [44–46].

Allen *et al.* [7] are also justified in their assertion that HRG does not, by itself, provide causal explanations of evolutionary change. Regression coefficients capture statistical associations, and statistical association is not causation [47–50]. To explain change causally, it is not enough to know the values of the population statistics *r*, *b* and *c*. These population statistics *mathematically imply* changes in gene frequency (just as the state-by-state breakdown mathematically implies the national result), but they do not *cause* those changes (any more than the state-by-state breakdown causes the national result). For a causal explanation, one also needs to know the evolutionary dynamics causally responsible for the values of *r*, *b* and *c*, including, where applicable, the population structure and the payoff structure of social interaction.

For example, positive relatedness (*r *> 0) in a population may be explained by underlying assortative processes as diverse as kin discrimination, limited dispersal, shared habitat preference, recognition of greenbeard-like phenotypic markers, or even, in microbes, gene mobility, and the way in which positive relatedness is generated can make a difference to the evolutionary stability of a social trait [32,35,41,51–54]. In any particular scenario in which relatedness is found to be positive, a satisfactory causal explanation of change should thus cite the causes of positive relatedness; if the aim is to elucidate the causal processes driving change, it is not enough to state merely that relatedness is positive without saying why. The same can be said of cost and benefit.

### 3.2. What the defenders get right

In spite of all this, I maintain that HRG is a useful and important result. This is because it provides an *organizing framework* that helps us structure our thinking about the causes of social evolution [55]. An organizing framework is not in competition with detailed models of particular ecological scenarios. It is intended to provide us with a helpful way of interpreting, classifying and comparing such models.

**Figure 1.**
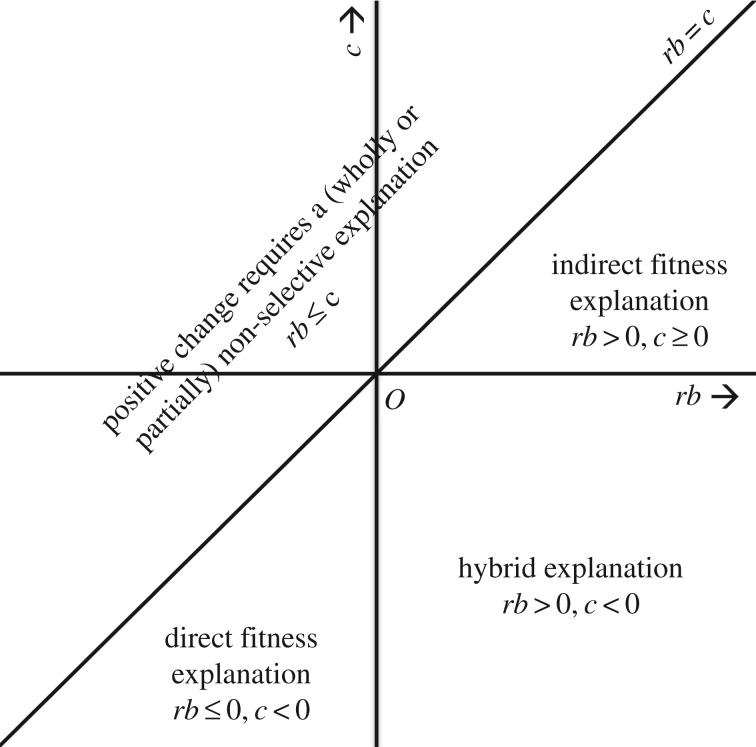
Partitioning the space of explanations. HRG allows us to distinguish four broad classes of explanation of positive evolutionary change in a social trait, defined by their commitments regarding the values of *rb* and *c*. All explanations of positive change lie somewhere in this space. The corresponding space for negative change is an inversion of this space (with *O* as the centre of inversion). (Reprinted from Birch [55] (Copyright © 2017, the author).)

The idea is that, by partitioning change into two biologically meaningful components (*rb* and –*c*), HRG provides an organizing framework that is distinctively valuable for social evolution research. What it does, in essence, is provide a scheme for categorizing causal explanations of evolutionary change. Any causal explanation of change in a particular trait, whether it takes the form of a detailed mathematical model or an informal verbal account, will have implied or explicit commitments regarding the costs and benefits of the trait and the relatedness between social partners. HRG provides a way of categorizing explanations by these commitments; for it shows that all causal explanations of positive change, in any character and in any population, must fall into one of four broad categories, depending on the signs of *rb* and *c*. These categories can be visualized in terms of a space of explanations (figure 1).

First, there are *indirect fitness explanations*, for which *rb* > 0 and *c* ≥ 0. These explanations cite a cause of relatedness to explain why the direct fitness costs associated with a trait (i.e. costs to the actor) are offset by indirect fitness benefits (i.e. benefits to related recipients). Second, there are *direct fitness explanations*, for which *c* < 0 and *rb *≤ 0. These explanations show how, over the lifetime of the actor, the trait yields direct fitness benefits, so that no indirect benefits are required. Third, there are *hybrid explanations* that appeal to both direct and indirect fitness effects as drivers of positive change, and for which *rb* > 0 and *c *< 0. Fourth, there are partially or wholly *non-selective explanations* that appeal to a process other than fitness differences between organisms. Such processes may include drift, migration, mutation, environmental change or forms of within-organism selection such as gametic selection or meiotic drive (formally, these are processes captured in the ‘transmission bias’ term in the Price equation and in Frank's ‘exact-total’ version of Hamilton's rule; [45,56,57]). HRG tells us that, if *rb* ≤ *c*, any adequate explanation of positive change must appeal to at least one such process.

The insight embodied by HRG is that every adequate causal explanation of positive change can be placed somewhere in this space. It also shows how the space of possible explanations is constrained by adding information about cost, benefit and relatedness. For example, if a trait is known to be costly in the technical sense that it detracts from the lifetime reproductive success of the actor (implying positive *c*), HRG tells us that an adequate causal explanation of positive change in that trait must appeal either to indirect fitness effects or else to non-selective processes, because direct fitness effects alone are not sufficient. These are insights that are obvious once one understands HRG, but far from obvious otherwise.

We can use this organizing framework as the basis for a more detailed taxonomy of explanations of change [48]. For example, indirect fitness explanations can be classified at a finer grain by the causes of relatedness they cite (e.g. kin discrimination or limited dispersal). Direct fitness explanations can be classified at a finer grain by the nature of the causal pathway that positively links the behaviour to the lifetime reproductive success of the actor, which may involve immediate returns, or may be mediated by reciprocity, punishment or reward. In this way, HRG structures the way social evolution theorists think about the causes of social evolution, shaping research programmes and allowing for overarching syntheses of diverse results [19,57–60].

Importantly, this organizing role of HRG is not undermined by the examples Allen *et al*. construct to challenge its predictive and causal-explanatory utility [7]. For instance, Allen *et al*. describe a scenario in which a ‘hanger-on’ trait causes its bearers to seek out and interact with individuals of high fitness. This leads to an association between an organism's fitness and the behaviour of its social partner, implying positive *b*, yet the hanger-on trait makes no causal contribution to fitness. Such examples show that one should be wary of interpreting *b* and *c* as measures of direct causal influence. However, they are not (and are not intended to be) counter-examples to HRG, and they do not show that HRG misclassifies explanations of change. The scenario Allen *et al*. describe is one in which *rb* = *c*, so the categorization scheme in figure 1 implies, correctly, that any positive change in the hanger-on trait must be explained by a non-selective process.

The best way to challenge the use of HRG as an organizing framework is not to construct counter-examples (because all sides agree that it is a correct mathematical result), but to argue that a different partition of change provides a more useful or biologically insightful categorization scheme [55]. Here, the defender of HRG should concede that it is certainly not the only possible organizing framework for social evolution research. The Price equation (from which HRG is derived) can be regarded as providing an organizing framework at an even coarser grain of analysis: it simply partitions change into a component due to natural selection and a component due to transmission bias, without partitioning the change due to natural selection into any further components [61–63]. HRG, by partitioning the change due to selection at a finer grain of analysis than the Price equation, is particularly useful for organizing explanations of social evolution. One notable rival to HRG in this respect is the framework of multilevel selection theory [32,56,64,65]. This too can be seen as a framework that organizes our thinking about the causes of social evolution by partitioning the space of explanations. This is not the place for a detailed comparison of the two frameworks (see [43,55]), but one important limitation of the multilevel framework, at least in the version developed by Price, is worth emphasizing: it only applies in populations that have a certain kind of structure, whereby the population is subdivided into objective, discrete and social groups. A distinctive advantage of HRG is that it still holds regardless of the population structure.

### 3.3. The way forward

HRG has been criticized for being an ‘empty statement’ or tautology, for failing to yield predictions of change, and for failing to yield causal explanations of change. There is some justification for all of these charges, but they do not undermine the use of HRG as an organizing framework: a framework for interpreting, comparing and classifying more detailed evolutionary models.

It is a virtue of an organizing framework that it operates at a high level of abstraction, invoking few assumptions: this makes it compatible with a wide range of underlying models, while also allowing us to make biologically meaningful comparisons between those models. For example, it allows us to see that, for all their underlying differences, models of the evolution of costly social behaviour by natural selection must invoke a cause of relatedness, such as kin discrimination, limited dispersal, shared habitat preference, greenbeard effects or gene mobility.

There is room for a productive debate regarding the value of HRG in comparison to other possible organizing frameworks, such as multilevel selection theory. Progress on this issue can be made by identifying the properties we value in an organizing framework (such as its compatibility with different possible population structures), and by evaluating the extent to which different frameworks possess these properties.

## 4. The status of inclusive fitness

Hamilton's rule is a statistical, population-level result. Inclusive fitness, by contrast, is an explicitly *causal* property of an *individual organism*. Here is how Hamilton defined inclusive fitness:

Inclusive fitness may be imagined as the personal fitness which an individual actually expresses in its production of adult offspring as it becomes after it has been stripped and augmented in a certain way. It is stripped of all components which can be considered as due to the individual's social environment, leaving the fitness he would express if not exposed to any of the harms or benefits of that environment. This quantity is then augmented by certain fractions of the quantities of harm and benefit which the individual himself causes to the fitness of his neighbours. The fractions in question are simply the coefficients of relationship. [30, p. 8]

**Figure 2.**
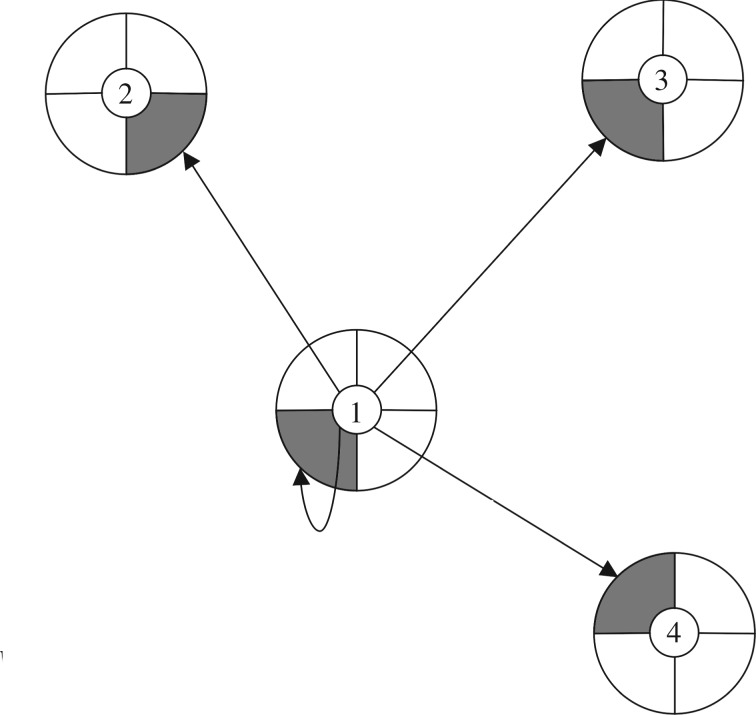
Inclusive fitness. An individual organism's inclusive fitness is a weighted sum of the effects of its behaviour on reproductive success. In this illustration, organism 1's behaviour affects the reproductive success of itself and of organisms 2, 3 and 4 (as shown by the arrows; the shaded regions represent components of reproductive success caused by the behaviour of organism 1). Organism 1's inclusive fitness consists of a baseline non-social component, plus the effect on its own reproductive success caused by its own behaviour, plus its effects on organisms 2, 3 and 4, weighted in each case by the relevant coefficient of relatedness. In a population without class structure, the coefficient of relatedness will be the same for every social partner and will correspond to the *r* coefficient in HRG (for discussion of cases in which class structure is present, see [39,45,46]). (Reprinted from Birch [55] (Copyright © 2017, the author).)

As noted above, there are other theorists who think about inclusive fitness differently: Queller, for example, has argued that we should think of inclusive fitness as a property of a trait or strategy rather than an individual [36], and Akçay & van Cleve argue that we should think of it as a property of a lineage [37]. But we can see clearly that, for Hamilton himself, inclusive fitness was, first and foremost, a property of an individual. We can, of course, talk of the mean inclusive fitness of a population, or of the bearers of a particular trait. But these averages are derivative notions: the fundamental notion is a property of an individual. It also could not be clearer that inclusive fitness, as Hamilton conceived it, is inherently causal: it is a weighted sum of the effects on reproductive success for which the focal organism is *causally responsible* (figure 2).

### 4.1. What the critics get right

Critics of inclusive fitness argue that it is committed, at a conceptual level, to the validity of an *additive causal model* of fitness [1,7]. They are right about this. The procedure Hamilton describes in the above paragraph involves crediting components of reproductive output to the actors whose social behaviour was causally responsible for them, rather than crediting them to the organisms that actually produced the offspring. For example, the larvae produced by a queen in a social insect colony should be credited not to the queen but to the workers who rear them. If this procedure is to avoid problems of double-counting, it must be that the reproductive success of an organism can be written as a *sum* of components, each of which is attributable to the social behaviour of a single social actor. Let us call this assumption *fitness additivity*. Moreover, it must be that the value of each component depends only on the genotype of the actor, and not on the genotype of the recipient, an assumption known as *actor's control* [39].

The assumptions of fitness additivity and actor's control are essential for inclusive fitness, conceived as a property of an individual organism, to make sense. Grafen, for example, writes that ‘the question of how to define inclusive fitness in the absence of additivity has not been settled, and so fundamental theory on the non-additive case can hardly yet begin’ [39, p. 544]. Thus, the reliance of inclusive fitness, as Hamilton conceived it, on fitness additivity and actor's control is something even its most committed defenders should acknowledge. Note here that, by contrast, the population-level result HRG has been applied to cases of non-additive payoffs. Regardless of the causal structure of social interaction, one can always use a regression model to partition change at the population level into an *rb* component and a −*c* component [17,33,42,43,59]. This, however, is not the same as defining inclusive fitness *qua* property of an individual organism in the non-additive case. Grafen rightly identifies this as a genuine problem.

The critics proceed to argue that there are many biologically plausible ways in which violations of these assumptions can arise. They are right about this too, although the point is not new: authors in the social evolution literature have made it repeatedly [66–69]. Consider, for example, a genotype that disposes its bearer to produce an alarm call. In so doing, it reveals the organism's location to nearby predators, adversely affecting its ability to benefit from the alarm calls of others. In this scenario, the benefit of receiving an alarm call for a recipient does not just depend on the genotype of the actor. It also depends on a fact about the recipient (i.e. whether or not the recipient has also produced an alarm call) that is sensitive to its genotype. Actor's control is violated.

Defenders of inclusive fitness should accept this too. Fitness additivity and actor's control are strong assumptions, and they are unlikely to be exactly true in real populations. In fact, Grafen does acknowledge this, writing, for example, that ‘the assumption of additivity is made throughout this paper, but is not in general a realistic assumption. In many applications, non-additivity is an important part of the problem’ [39, p. 543]. The critics may reply, with some justification, that this point is absent from some of the more forthright defences of inclusive fitness [11].

### 4.2. What the defenders get right

Critics of inclusive fitness are likely to feel that the discussion should end here: because inclusive fitness makes strong assumptions that are often violated in real populations, we should stop using it as a fitness concept. For example, Allen & Nowak [9] conclude from the unrealistic nature of the additivity assumption that ‘there is no inclusive fitness at the level of the individual’. Yet, despite all of the above, I think a good case can be made for the theoretical value of inclusive fitness, conceived as a property of an individual.

#### 4.2.1. Weak selection and Fisher's microscope

Defenders of inclusive fitness have often noted that its assumptions can be justified as *approximations* if we assume a specific form of weak selection, usually known as *δ*-weak selection [39,70–72]. To assume *δ*-weak selection is to assume that the character of interest is a quantitative character, and that the alternatives competing in the population are a wild-type and a mutant that differs only very slightly from the wild-type. For example, a *δ*-weak selection model of an alarm call scenario might pit a wild-type strategy in which an organism makes an alarm call with probability *q* against a mutant strategy in which an organism makes an alarm call with probability *q* + *δ*, where *δ* is a very small increment such that *δ*^2^ ≈ 0 [72].

With this assumption in place, we can reinterpret inclusive fitness in terms of *marginal* (or *differential*) causal effects, attributable to small deviations from the wild-type, rather than *total* causal effects. In other words, instead of defining an actor's inclusive fitness as a baseline component plus a weighted sum of the total effects of its behaviour on reproductive success, we instead define an actor's inclusive fitness as a baseline component plus a weighted sum of the *differential* effects of its behaviour on reproductive success *relative* to a default scenario in which the actor expresses the wild-type behaviour. All effects common to the mutant and wild-type are thus folded into the baseline component of fitness. On this marginal interpretation, fitness additivity and actor's control can be reinterpreted as assumptions about marginal effects: what is assumed is that the marginal effects of the mutant phenotype, relative to the wild-type, are additive and actor controlled.

The upshot is that the assumptions that initially seemed too strong are now reasonable as approximations. To see the intuitive rationale for this, consider again the alarm call example. The problem here was that making an alarm call reduces the benefit an organism receives from an alarm call expressed in others, leading to a violation of actor's control. But now consider the marginal effect of making an alarm call with probability *q* + *δ* rather than probability *q*. This will have a first-order effect (proportional to *δ*) on one's own reproductive success and on the reproductive success of nearby recipients. It will also have a second-order effect (proportional to *δ*^2^) on the benefit one receives from a very small increase in the probability with which another nearby individual makes an alarm call. However, this second-order effect, which is the source of the trouble for the actor's control assumption, is precisely the kind of effect that the assumption of *δ*-weak selection entitles us to regard as approximately 0, because it relies on the product of two tiny phenotypic differences.

To the critics, the appeal to *δ*-weak selection here will seem *ad hoc*: to justify two questionable assumptions, we invoke another assumption that seems no less questionable [1,7]. Why think that selection is usually *δ*-weak? Why think it is ever *δ*-weak? However, I do not see this as an *ad hoc* assumption. It is fairer, I think, to see it as an assumption grounded in some important background commitments of inclusive fitness theory—commitments that can be traced back to Hamilton, but which critics of inclusive fitness do not necessarily share.

At the heart of the Hamiltonian tradition is a version of adaptationism that takes complex adaptation, or ‘organism design’, to be the explanatory target of social evolution research [40,73]. This is combined with an empirical commitment to a gradualist picture of how complex adaptation arises. Fisher, a major influence on Hamilton, took complex adaptation to result from the gradual accumulation of mutations with tiny phenotypic effects (Darwin was also a gradualist, but the picture of adaptation arising through the accumulation of small-effect mutations is properly credited to Fisher) [74]. Fisher posited small-effect mutations on the grounds that large-effect mutations are much less likely to cause adaptive improvements. In support of this, he offered two iconic arguments: one involving an informal analogy with a microscope and the other involving a more formal geometric model.

To paraphrase (and simplify) the informal argument, suppose you are attempting to focus a microscope by turning an adjustment knob. Knowing nothing of microscopes, you have no idea which way to turn the knob, so you turn it in a random direction. If the adjustment is very small, there is a 50% chance it will improve the focus, because any very small adjustment in the right direction will help. But the larger the adjustment gets, the lower the probability it will be an improvement, because it becomes ever more likely that an adjustment, even if it happens to be in the right direction, will overshoot the target.

Using a geometric model in which a population is displaced from the optimum in phenotypic space and must find its way back to the optimum through random gene substitutions, Fisher showed that the probability of an improvement, which falls off with the size of the adjustment even in the one-dimensional case, falls off more rapidly in the case of an adjustment in two dimensions and falls off very rapidly indeed when we are adjusting at random in many dimensions, as in the case of a mutation that affects many aspects of the phenotype. The chance of improvement is greatest, at 50%, for a mutation that affects the phenotype by an infinitesimal amount.

Fisher's argument has not been without its critics. Kimura argued that, in finite populations, mutants with larger effects on the phenotype have a greater chance of going to fixation, because mutants with small effects are prone to drifting out of existence [75]. Orr showed that both Fisher and Kimura could be partially vindicated in relation to different stages of the process of cumulative adaptation: the typical effect size of a mutation fixed at an early stage in the process, when the phenotype is far from the optimum, is much larger than Fisher thought; but, as the phenotype gets closer to optimality, Fisher's concern about overshooting becomes increasingly salient and the typical effect size of a fixed mutation becomes progressively smaller [76].

Although Fisher's argument remains a source of debate [77,78], what matters for our purposes is that a commitment to Fisherian gradualism is at the heart of Hamilton's theory of social evolution. Consider, for example, the following *credo* from Hamilton's collected papers:

I was and still am a Darwinian gradualist for most of the issues of evolutionary change. Most change comes, I believe, through selected alleles that make small modifications to existing structure and behaviour. If one could understand just this case in social situations, who cared much what might happen in the rare cases where the gene changes were great and happened not to be disastrous? Whether under social or classical selection, defeat and disappearance would, as always, be the usual outcome of genes that cause large changes. I think that a lot of the objection to so-called ‘reductionism’ and ‘bean-bag reasoning’ directed at Neodarwinist theory comes from people who, whether through inscrutable private agendas or ignorance, are not gradualists, being instead inhabitants of some imagined world of super-fast progress. Big changes, strong interlocus interactions, hopeful monsters, mutations so abundant and so hopeful that several may be under selection at one time—these have to be the stuff of their dreams if their criticisms are to make sense. [79, pp. 27–28]

Thus, a focus on *δ*-weak selection is grounded in the core commitments of Hamilton's programme. The subset of selection processes for which inclusive fitness is a valid fitness concept is the same subset Hamilton and his successors take, on independent grounds, to be responsible for the cumulative assembly of complex adaptations.

#### 4.2.2. Inclusive fitness as a criterion for improvement

There is more to be said, however, about the connection between inclusive fitness and cumulative adaptive evolution. The fact that inclusive fitness is a valid fitness concept under *δ*-weak selection does not give it any advantage over other valid fitness concepts. But, in the context of explaining cumulative adaptation, there is a different theoretical role for a fitness concept with respect to which inclusive fitness is distinctively valuable: that of providing a *criterion for phenotypic improvement*.

Suppose we are trying to explain the evolution of a complex adaptation through the gradual accumulation of tiny improvements to the phenotype. Talk of improvement implies a standard with respect to which improvement can be judged. In this context, we want more from a fitness concept than accurate calculations of short-term gene frequency change and short-term equilibria. We also want a fitness concept that can provide a stable criterion, throughout the whole process, for what constitutes an improvement to the phenotype. In other words, we want a property of an organism, *X*, such that new mutants are systematically favoured over the wild-type if and only if they make a positive causal contribution (in contrast with the wild-type) to *X.* The distinctive advantage of inclusive fitness over other fitness concepts is that it is a good candidate for property *X*.

To see why, imagine a process of social evolution in which natural selection gradually shapes various different aspects of a complex social strategy involving the conditional expression of different actions in different contexts. In one context, *C*_1_, the strategy produces actions that benefit the actor; in another context, *C*_2_, the strategy produces actions that confer benefits on genetically related recipients. Mutants periodically arise (one at a time) that alter some aspect of the strategy very slightly, implying *δ*-weak selection.

Suppose natural selection targets different aspects of the phenotype at different times: the strategy is initially shaped by selection for enhanced benefits for the actor in *C*_1_, then goes through a stage in which it is shaped by selection for greater benefits conferred on genetically related recipients in *C*_2_, and then finally goes through a streamlining stage in which the cost to the actor of conferring benefits on relatives in *C*_2_ is gradually reduced. A more realistic scenario would involve the shaping of all these aspects of the phenotype as and when relevant mutants arise; but, for the purpose of fixing ideas, it helps to think of selection targeting different aspects in discrete stages.

At all stages in this hypothetical process, the actor's inclusive fitness provides a criterion for improvement: all and only those mutants that causally promote (in contrast to the wild-type) the inclusive fitness of the actor are favoured. The same cannot be said of the actor's reproductive success, because mutants that detract from this quantity are favoured during the middle stage; nor can it be said of the recipient's reproductive success, because mutants that may be neutral or deleterious with respect to this quantity are favoured in the initial and final stages.

The cumulative assembly of social adaptations through *δ*-weak selection thus constitutes a special context in which inclusive fitness is both valid and valuable. It is valid because its assumptions are reasonable as approximations when selection is *δ*-weak. It is valuable because, unlike other fitness concepts, it provides a stable criterion for what constitutes an improvement as natural selection shapes different aspects of the phenotype.

### 4.3. The way forward

The assumptions of inclusive fitness are empirically questionable if interpreted as exact claims about total effects. However, they can be justified as approximations regarding marginal effects under *δ*-weak selection, which is to say selection on tiny differences between the mutant and the wild-type. The assumption of *δ*-weak selection is grounded in a methodological stance that takes complex adaptation to be the explanatory target of social evolution research, together with an empirical commitment to a gradualist picture on which complex adaptation arises through the accumulation of small improvements. In the context of a process of cumulative adaptive evolution by *δ*-weak selection for small improvements, inclusive fitness has a distinctive role to play as the criterion for improvement.

## 5. Selection, design and optimality

This special issue provides an opportunity to reflect not only on the mathematical foundations of inclusive fitness theory, but also on its connections to questions of purpose, design and optimality in the natural world. Here too, we find that the two opposing camps in the current controversy hold radically different views.

### 5.1. One extreme

Defenders of inclusive fitness are, for the most part, *adaptationists* who regard natural selection as a powerful generator of phenotypic optimality. Here, we should distinguish two types of adaptationism. We noted above that inclusive fitness theorists take complex adaptation (or ‘organism design’) to be their main explanatory target. Because this is a methodological choice, rather than a claim about nature, it is a version of *methodological* adaptationism [80,81]. This should be distinguished from the claim that natural selection has a robust tendency to generate phenotypic optimality, and that we should therefore expect organisms to behave as if at least approximately optimizing their inclusive fitness. This is a claim about nature, and it is therefore a version of *empirical* adaptationism [80,81].

I am sceptical of the second, stronger form of adaptationism [82,83]. The issue is a complex one, but for current purposes it is sufficient to note that the ability of natural selection to produce cumulative improvement depends on many variables, including the stability of the environment, the strength of other evolutionary causes such as drift and the genetic architecture underlying the trait [84–86]. Although social evolution researchers like to play the ‘phenotypic gambit’ and assume that the genetic basis of social traits is simple and conducive to adaptation [87], we must remember that this is a gambit—an opening bet—and not a well-supported empirical assumption. Genetic details often turn out to matter, and there are many reasons why a process of cumulative improvement may stall, or never get off the ground at all [82,83]. There is no reason to assume that these variables are generally favourable to cumulative improvement in natural populations, and there is no substitute for testing the underlying assumptions of optimality models empirically [88,89].

Note, however, that merely regarding inclusive fitness as a *criterion* for improvement does not imply any commitment to empirical adaptationism. We are saying here that inclusive fitness provides the bar against which the improvement or degradation of a trait should be judged. This does not imply that natural selection will always, often, or indeed ever succeed in generating cumulative improvement, let alone optimality, in the natural world. The question of how often this happens is not a question theory alone can settle [82,83].

Inclusive fitness optimization should not, therefore, be taken as an unchallenged foundation for projects in behavioural ecology. It should be regarded as an empirical conjecture that may hold in some populations some of the time, but not in all populations all of the time. I do not think this concession undermines inclusive fitness theory in any significant way. It should, however, spur us to investigate the special features of those ‘paradigm Darwinian populations' (in Godfrey-Smith's terminology) in which cumulative improvement does reliably occur [86].

### 5.2. Another extreme

Allen *et al.* [7] write that ‘There is no universal design principle … [and] no universal maximands or design principles are needed to understand the evolution of social behavior’. If the intended emphasis is on the term *universal*, and if Allen *et al.* are happy to grant a role for design principles with circumscribed domains of application, I find little to disagree with here. But I suspect they are expressing a broader scepticism about the use of ‘design thinking’ in social evolution research—that is, scepticism about the general strategy of attempting to understand behaviour by looking for a ‘design objective’ the behaviour promotes.

By contrast, I maintain there is a legitimate, though circumscribed, role for design thinking in the study of social evolution. The gradualist's dictum, common to Darwin, Fisher and Hamilton, is that, when we find a complex adaptation, we should infer that it has been produced by a process of cumulative adaptive evolution in which small incremental improvements were favoured, and not by the sudden appearance of a hopeful monster followed by a single-step selection process. But note that the concept of *improvement* is indispensable to an understanding of this process: the fact that natural selection systematically favours *improvements* to the phenotype is what makes the process *cumulative* and the end products *adaptive*. Note, moreover, that the notion of improvement is unintelligible without a standard with respect to which improvement is to be judged, and that inclusive fitness provides the appropriate standard (§4). To be a gradualist is therefore to acknowledge a place in evolutionary biology for a limited form of design thinking, based around the concept of improvement, and a legitimate place for inclusive fitness as the fitness concept with respect to which an improvement is defined.

### 5.3. The way forward

There is a special type of evolutionary process—the gradual assembly of complex adaptation through the accumulation of small improvements—with respect to which a form of design thinking is appropriate. In this special context, as noted in §4, inclusive fitness has a special role as the criterion for improvement. This is not to endorse a universal design principle. Theory alone cannot tell us how often, if ever, natural selection leads to cumulative improvement, let alone optimality, and such a process is not to be expected in all populations at all times. However, the existence of complex adaptations is not in doubt, and this indicates that natural selection has succeeded in generating cumulative improvement in at least some cases. These cases hold a special (and understandable) fascination for behavioural ecologists in the Hamiltonian tradition, which helps explain and justify their continuing attachment to the concept of inclusive fitness.

## 6. Conclusion

In attempting to reconcile the two camps in the inclusive fitness controversy, I hope to have arrived at a synthesis that does justice to the insights of both sides, and not merely at an awkward compromise. Hamilton's rule in its regression-based form—a statistical, population-level result—has genuine predictive and explanatory limitations, but it remains valuable as an organizing framework for social evolution research. There is room for a productive debate regarding its relative value in comparison to other possible organizing frameworks, such as multilevel selection theory; and progress on this issue can be made by identifying the properties we value in an organizing framework and by evaluating the extent to which different frameworks possess these properties.

Meanwhile, inclusive fitness—which, for Hamilton, was a causal property of an individual organism—relies on the assumptions of fitness additivity and actor's control. These assumptions are reasonable approximations given a specific type of weak selection. There is room for productive debate about whether (as the critics suggest) weak selection represents a far-fetched case of little biological interest, or whether (as Hamilton and his defenders would have it) it is the process through which complex adaptation is cumulatively assembled. If the latter is correct, then inclusive fitness has a special role to play as a criterion for improvement, and this licenses a limited form of design thinking.

This short paper has not aimed to settle these debates. But I hope to have shown that the differences between the two sides are not irresolvable, and that productive debate on these issues is possible.

## Data accessibility

There is no supporting data associated with this paper.
